# Bone marrow-derived progenitor cells attenuate inflammation in lipopolysaccharide-induced acute respiratory distress syndrome

**DOI:** 10.1186/1756-0500-7-613

**Published:** 2014-09-08

**Authors:** Neysan Rafat, Christine Dacho, Gregor Kowanetz, Christian Betzen, Burkhard Tönshoff, Benito Yard, Grietje Beck

**Affiliations:** Department of Pediatrics I, University Children’s Hospital Heidelberg, University of Heidelberg, Heidelberg, Germany; Clinic for Anaesthesiology and Critical Care Medicine, Alfried-Krupp-Krankenhaus, Essen, Germany; Department of Radiology, Minimally Invasive Therapies and Nuclear Medicine, SLK Kliniken Heilbronn GmbH, Heilbronn, Germany; Division of Functional Genome Analysis, German Cancer Research Center (DKFZ), Heidelberg, Germany; Department of Medicine V, Mannheim University Medical Center, University of Heidelberg, Heidelberg, Germany; Department for Anaesthesiology and Intensive Care Medicine, Dr. Horst-Schmidt Clinic, Wiesbaden, Germany

**Keywords:** Adult stem cells, Bone marrow-derived progenitor cells, Cell therapy, Acute respiratory distress syndrome, Lipopolysaccharide

## Abstract

**Background:**

Acute respiratory distress syndrome (ARDS) is the most common cause of respiratory failure among critically ill patients. Novel treatment strategies are required to address this common clinical problem. The application of exogenous adult stem cells was associated with a beneficial outcome in various pre-clinical models of ARDS. In the present study we evaluated the functional capacity and homing ability of bone marrow-derived progenitor cells (BMDPC) *in vitro* and investigated their potential as a treatment strategy in lipopolysaccharide (LPS)-induced ARDS.

**Results:**

Evaluation of the BMDPC showed functional capacity to form endothelial outgrowth cell colonies, which stained positive for CD133 and CD31. Furthermore, DiI-stained BMDPC were demonstrated to home to injured lung tissue. Rats treated with BMDPC showed significantly reduced histopathological changes, a reduced expression of ICAM-1 and VCAM-1 by the lung tissue, an inhibition of proinflammatory cytokine synthesis, a reduced weight loss and a reduced mortality (p < 0.03) compared to rats treated with LPS alone.

**Conclusions:**

These findings suggest that the application of exogenous BMDPC can attenuate inflammation in LPS-induced ARDS and thereby reduce the severity of septic organ damage. Cell therapy strategies using adult stem cells might therefore become a novel and alternative option in ARDS therapy.

## Background

Acute respiratory distress syndrome (ARDS) is the most common cause of respiratory failure among critically ill patients. It commonly develops in patients with sepsis and in this setting associates with high mortality [1]. Regardless of the cause, the histologic changes of lung tissue in the course of ARDS are characterized by infiltration of inflammatory cells and destruction of pulmonary endothelium [2, 3] leading to increased pulmonary vascular permeability and edema. Despite extensive research and well-conducted clinical trials, no specific novel treatment strategy exists to date [4, 5] and existing therapy is currently limited to supportive care [6, 7]. A novel potential treatment strategy for ARDS is the use of bone marrow-derived progenitor cells (BMDPC) [8–11]. In this regard, especially endothelial progenitor cells (EPC) and mesenchymal stem cells have been shown to play a role. An increased concentration of EPC was found in septic patients, which seem to be involved in pulmonary regeneration. The number of circulating EPC correlated inversely to disease severity and mortality [12]. For a detailed overview on the role and effect of EPC in regeneration after acute lung injury (ALI), please see [13]. While EPC initially were thought to be recruited and incorporated into sites of active neovascularization during e.g. tissue ischemia, vascular trauma, tumor growth and inflammation [14], more recent work suggests different populations of endothelial progenitor cells with distinct functions [13].

A vast amount of pre-clinical studies have been conducted to investigate the potential of mobilization or administration of BMDPC in lung regeneration in different lung disorders [13]. However, it is still unclear whether progenitor cells can beneficially influence regeneration of ARDS and which cell population should be used. We have previously demonstrated that the application of BMDPC in an endotoxin-induced ARDS rat model lead to an improved gas exchange, an inhibition of proinflammatory cytokine synthesis, an improved clinical course and a reduced mortality [15]. However, we have not looked at the functional capacity of the administered BMDPC to prove that they are responsible for the improved clinical course and reduced mortality. Therefore in the present study, we investigated in our isolated endotoxin-induced ARDS rat model on one hand whether exogenically administered bone marrow-derived CD133^+^ progenitor cells could beneficially improve inflammation and survival. On the other hand, we evaluated the functional capacity and homing ability of the BMDPC, which were applied in our model.

## Results

### Animal model

**Figure 1:**
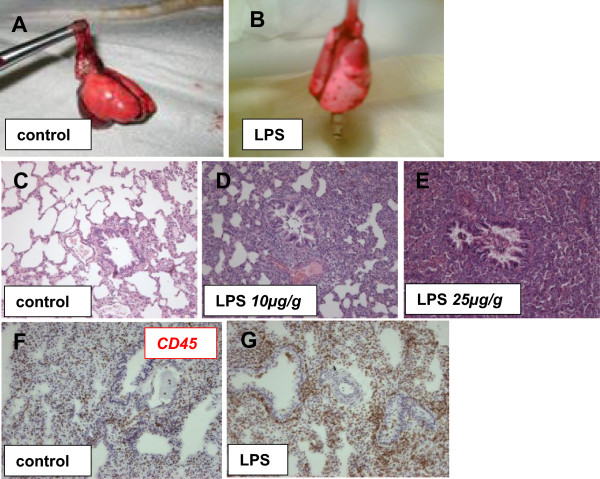
**LPS-induced ARDS in a rat model.** The macroscopic changes in lung seventy-two hours after lipopolysaccharide (LPS) nebulization **(B)** and in control animals are displayed **(A)**. The lungs of animals receiving LPS were infiltrated in large or disseminated in small areas, to some extent livid discolorations and hemorrhages were visible. Lung tissue damage could also be determined immunhistochemically. While the control group **(C)** showed intact lung tissue, LPS led to thickening of the alveolar wall **(D, E)** and an accumulation of inflammatory cells. CD45 staining showed increased numbers of leucocytes in animals treated with LPS **(G)** compared to those undergoing a sham operation **(F)**.

The results of an extensive dose–response analysis revealed reproducible lung damage with distinct clinical symptoms consistent with moderate-to-severe ARDS at a LPS-concentration of 25 μg/kg of body weight. The lungs of animals receiving LPS were infiltrated in large or disseminated in small areas, to some extent livid discolorations and hemorrhages were visible (Figure 1A and 1B). Histopathologically, LPS-nebulization led to thickening of the alveolar wall (Figure 1D and 1E) and an increased accumulation of CD45 + −leucocytes (Figure 1G) compared to the control group (Figure 1C and 1F). The number of animals in the individual groups was as follows: control group: n = 9, treatment group: n = 17, non-treatment group: n = 17.

### Endothelial Outgrowth Cell (EOC) Assay

**Figure 2:**
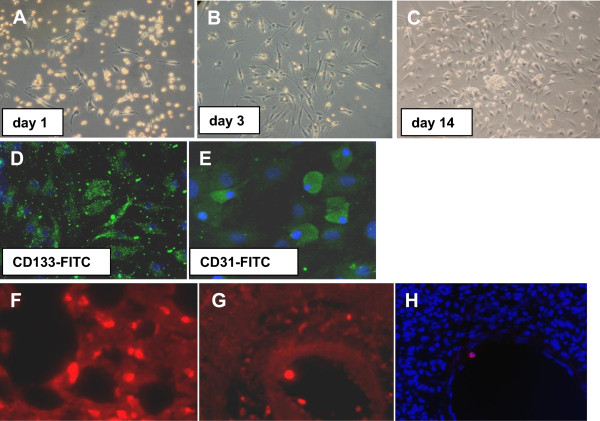
**Isolation, characterization and homing of bone marrow-derived progenitor cells (BMDPC).** BMDPC were isolated from the bone marrow cell suspension by positive selection for CD133 by microbeads associated with monoclonal anti-CD133-antibodies. To test their functional capacity, the cells were seeded in culture flasks for 3 weeks. They were able to form endothelial outgrowth cell colonies quite early **(A,B)** and proliferated to a high level of confluence **(C)** as seen via phase contrast microscopy. Staining for CD133 and CD31 showed that the cultured cells adopted an endothelial character **(D,E)**. To investigate the homing process, BMDPC were stained with DiI and administered directly after LPS injection into the animals. After forty-eight hours the animals were sacrificed and their lung tissue analysed. Both in the alveolar tissue **(F)** and adhering to vascular wall **(G)** fluorescent cells could be detected. In the cryosection **(H)** nuclei are stained with DAPI and one fluorescent could be detected.

To test the functional capacity of the BMDPC, we seeded the cells in culture flasks for 3 weeks. The cells showed a good capacity to form endothelial outgrowth cell colonies and proliferate to a high level of confluence (Figure 2A-C). To verify their endothelial lineage, positive staining for CD133 and CD31 showed that the cultured cells adopted an endothelial character (Figure 2D and 2E).

### BMDPC homing assay

To investigate whether the BMDPC are able to home to injured lung tissue, BMDPC stained with DiI were administered directly after LPS injection into the animals. After forty-eight hours the animals were sacrificed and their lung tissue analysed. Both in the alveolar tissue (Figure 2F) and adhering to vascular wall (Figure 2G and 2H) fluorescent cells could be detected.

### Mortality

In the treatment group 13 out of 17 animals (76,5%) survived after 72 hours compared to 8 out of 17 animals (47,1%) in the non-treatment group. Therefore, the probability of survival of the animal in the treatment group is significantly increased compared to the non-treatment group (p < 0.03).

### Clinical symptoms

For the evaluation of the disease course, we chose in this study to observe the weight changes of the animals over 72 h. At the final time point the animals in the non-treatment group showed a weight loss of 15% compared to their initial weight, whereas the animals in the treatment group only lost 11% of their initial weight.

### Histopathologic data

**Figure 3:**
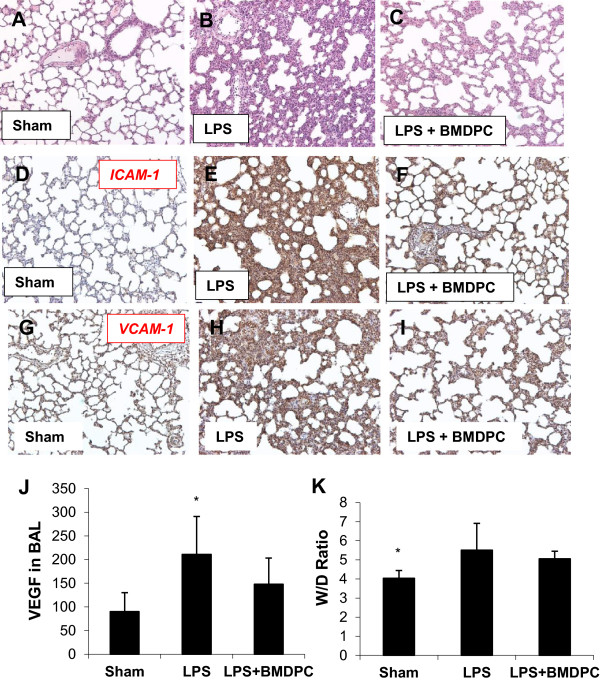
**Bone marrow-derived progenitor cells (BMDPC) attenuate inflammation.** Rats treated with BMDPC showed significantly improved lung tissue using HE staining **(C)** compared to non-treatment group **(B)**. Also, the expression of the proinflammatory adhesion molecules ICAM-1 **(F)** and VCAM-1 **(I)** were significantly reduced compared to the non-treatment group **(E, H)**. A sham group served as a control group for all stainings **(A, D, G)**. In the bronchoalveolar lavage (BAL) VEGF was significantly increased in the non-treatment group compared to the treatment and sham operation group **(J)**. The W/D Ratio did not reveal any significant differences between the treatment and the non-treatment group, while it was significantly lower in the sham group compared to the two other groups **(K)**; *p < 0,05 (values displayed as mean) non-treatment (LPS) versus treatment group (LPS + BMDPC).

Histopathologic evaluation revealed reduced interstitial acute inflammation, inflammatory cellular infiltration and thickening of the alveolar wall in the treatment group compared to the non-treatment group (Figure 3A-C). Also, the expression of the pro-inflammatory adhesion molecules ICAM-1 (Figure 3D-F) and VCAM-1 (Figure 3G-I) were significantly reduced in the treatment group compared to the non-treatment group.

### Bronchoalveolar Lavage (BAL)

Since inflammation triggers many cells to release VEGF, we measured the concentration of VEGF in the brochoalveolar lavage (BAL) at the final time point. In the treatment group VEGF was significantly decreased compared to the non-treatment group (Figure 3J).

### Wet/dry weight ratio

Pulmonary edema was analyzed by wet/dry weight ratio (W/D Ratio) of the lungs at the final time point. While the W/D Ratio in the control group was significantly decreased compared to the treatment and non-treatment groups (Figure 3K), there was no significant statistical difference between the treatment and the non-treatment group (Figure 3K).

## Discussion

Our findings demonstrated that exogenously administered BMDPC can attenuate inflammation in LPS-induced ARDS. Rats treated with BMDPC showed significantly reduced histopathological changes, a reduced expression of ICAM-1 and VCAM-1 by the lung tissue and a reduced concentration of proinflammatory VEGF in BAL. Moreover, the animal in the treatment group showed a reduced weight loss and mortality compared to rats treated with LPS alone. Evaluation of the BMDPC, which were applied in this study, showed functional capacity to form endothelial outgrowth cell colonies, which stained positive for CD133 and CD31. We were also able to demonstrate homing of DiI-stained BMDPC to injured lung tissue, but which was rather a seldom event. Previously, we have already demonstrated that the application of BMDPC in the same ARDS rat model lead to an improved gas exchange, an inhibition of proinflammatory cytokine synthesis, an improved clinical course and a reduced mortality [15]. However, we have not looked at the functional capacity of the administered BMDPC to prove that they are responsible for the improved clinical course and reduced mortality. Therefore, with the present study, we have added new insights into the therapeutic effects of BMDPC in our ARDS model.

It is undisputed among researchers within the field that BMDPC migrate to the location of revascularisation, initiating proangiogenic effects via paracrine signalling or in rather a small number differentiating directly to mature endothelial or other cell types [13]. Increasing significance is also attached to these progenitor cells with respect to endothelial cell regeneration after acute and chronic inflammation-induced tissue damage [16]. We and others have demonstrated in previous studies that progenitor cells are increasingly mobilized from the bone marrow into the circulation during sepsis [12], ARDS [17] and other inflammatory lung diseases [18].

The concentration of progenitor cells in the circulation also correlated with the clinical outcome of the patients. Based on this, a number of animal studies have been performed to evaluate the effect of BMDPC mobilization or administration in lung regeneration. Many of these studies used an endotoxin-induced model of ALI/ARDS. We have recently demonstrated that the application of BMDPC in the same LPS-induced ARDS rat model lead to an improved gas exchange, an inhibition of proinflammatory cytokine synthesis, an improved clinical course and a reduced mortality [15]. For a detailed overview of the *in vitro*-, clinical and animal studies looking at the role and the therapeutic effect of BMDPC in ALI/ARDS, please refer to [13].

In this present study, we examined on the one hand whether the exogenic application of BMDPC has a beneficial effect on the inflammation in experimental LPS-induced ARDS and thereby on the clinical course. On the other hand, we analysed whether the BMDPC population applied in this study show functional capacity in vitro and also home to the injured lung tissue.

We were able to demonstrate reduced histopathological changes, a reduced expression of ICAM-1 and VCAM-1 by the lung tissue, a reduced concentration of VEGF in BAL, a reduced weight loss and mortality in animals which were treated with BMDPC after LPS nebulization. Thus, the disease course was significantly improved. In our vulnerable *in vivo* model, LPS was nebulized only once to observe the acute phase of ARDS for seventy-two hours. For long-time observations, multiple or permanent applications of LPS will certainly have to be considered and evaluated. In this study, the animals receiving LPS only, who survived the acute stadium after seventy-two hours, remained stable and reconstituted fully.

Our observations confirm previous results of animal studies [19–24]. Gao *et al.* have demonstrated in a LPS-induced ARDS rabbit model that systemic application of progenitor cells inhibited the expression of adhesion molecules and the synthesis of pro-inflammatory cytokines (TNF-α or IL-1b) while significantly increasing the synthesis of anti-inflammatory cytokines (IL-10) [25]. In our study, we too observed a significantly reduced expression of adhesion molecules following administration of BMDPC. Interestingly, Gao *et al.* were also able to show a significant reduction of the inflammation-induced apoptosis of endothelial and epithelial cells by the application of progenitor cells [25]. Based on these observations, it seems that BMDPC not only stimulate vessel regeneration and repair but also inhibit local inflammation [26]. In a recent study, Rojas *et al.* have demonstrated a decrease of the severity of LPS-induced ARDS in sheep by the instillation of BMDPC [27]. Sheep treated with BMDPC showed rapid recovery in oxygen levels, carbon dioxide clearance, pulmonary vascular pressures and inflammation, confirmed by histology and by the decrease in lung edema [27], which we could not observe in our study. In another recent study, Fan *et al*. showed that the application of EPC and a SDF-1α analogue synergistically improved survival in CLP-induced sepsis in a mice [28]. In accordance with our observations, EPC-application resulted in decreased lung vascular leakage and reduced inflammatory cell infiltration in the lung [28].

The mechanisms involved in the beneficial effects of BMDPC/EPC are still unclear for the most part. Recruitment and incorporation of BMDPC/EPC from bone marrow into ischemic or injured tissue sites requires a coordinated multistep process including mobilization, chemotaxis, adhesion to the endothelium, transendothelial migration, invasion and in situ differentiation [13]. The transdifferentiation of BMDPC/EPC into mature endothelial cells is rather a rare event. Therefore, it was not unexpected that we could only find a small number of DiI-stained BMDPC homing to injured lung tissue In most cases BMDPC/EPC, after invasion into the subendothelial layer, release growth factors which stimulate angiogenic activity of resting mature endothelial cells [13]. This was also confirmed in a single-lung induced ARDS model after intravenous BMDPC application [22]. BMDPC homed exclusively to the lung with damaged endothelium, but not to the contralateral healthy lung or to any other healthy organ. Nonetheless, the experimental models were not able to confirm an *in vivo* differentiation of BMDPC into mature endothelial cells [22]. In our observations, we could also show homing of BMDPC to injured lung tissue and especially to the vessel wall. However, we looked only into the *in vitro* differentiation of BMDPC into mature endothelial cells. In a different study, BMDPC did not integrate into the vessel wall in ischemic models, but colonized in subendothelial layers or on the surface of the endothelium to induce their effects in a paracrine manner [29]. These paracrine effects consist of an increased release of growth factors as well as chemokines and cytokines. Thus, a proangiogenic environment is established, by which resident endothelial and other cell groups are stimulated to proliferate [30].

First results on the potential significance of the BMDPC surface molecules α4- or α5-integrin involved in the adhesion of the endothelial matrix have been published [24]. In an endotoxin-induced lung vascular injury and edema model in mice, 80 – 90% of the injected BMDPC could be found in the damaged pulmonary tissue within twenty minutes. But the concentration of the BMDPC decreased by 40% over the next twenty-four hours. Furthermore, it was also confirmed that adhesion of BMDPC to the tissue is required to induce the BMDPC-triggered reduction of vessel damage, oedema formation and mortality after LPS-induced inflammation. The therapeutic effects of BMDPC were not detectable anymore when the integrins were blocked and by that adhesion of BMDPC diminished [24].

BMDPC/EPC are characterized and isolated by the presence of trans-membrane glycoprotein CD133, which is expressed as surface antigen on human progenitor cells, but also on human hemangioblasts [31]. In contrast to the hematopoietic progenitor marker CD34, CD133 is not expressed on mature endothelial cells, and sub-populations of CD34^+^ cells, which also express CD133, have a high proliferative capacity and lead to the formation of endothelial colonies in cell culture [31]. We also observed that CD133^+^/BMDPC had a high proliferative capacity and lead to the formation of endothelial colonies, which were also CD34^+^. Studies in animal models report that circulating CD133^+^/BMDPC are involved in the neoangiogenesis after tissue ischemia and in the regeneration of the damaged organ [32, 33].

An increase of the BMDPC concentration in blood, as a therapeutical option for severe inflammation with endothelial damage in ARDS/ALI, may be induced in two manners. First, an endogenic mobilization of EPC from the bone marrow can be stimulated by administration of growth factors, such as VEGF, GM-CSF, EPO or SDF-1. By this means, an effect could only be documented in a single cardiological study [34, 35] so far, and results from our own experimental series in our LPS-induced isolated ARDS rat model are still pending. Second, the exogenic application/transplantation of BMDPC, as carried out in this study, seems to cause significant effects in the inflammation model, but comprises the disadvantage of requiring previous BMDPC preparation. In addition, differences in the treatment of local vessel damage with “on-site application” as opposed to treatment of generalized microvascular damage with systemic BMDPC application have to be taken into account.

There are different limitations to this study. We have used an isolated ARDS-model to investigate the role of BMDPC. Patients suffering ARDS on intensive care units are usually characterized by multi-morbidity, which has a strong impact on their disease course and treatment. Also, other factors affect the course of ARDS (e.g. different mechanical ventilation protocols, differences in PEEP application), which makes it difficult to transfer our findings to the clinical setting. Furthermore, we have administered BMDPC directly after LPS injection, which is not possible in a clinical setting. By the time ARDS is present, there might be no therapeutic effects of BMDPC anymore.

## Conclusions

In endotoxin-induced pulmonary endothelial dysfunction, BMDPC seem to initiate repair mechanisms, which result in a reduction of increased inflammation, permeability and oedema formation, thus reducing mortality. Future studies will have to analyse the underlying mechanisms involved in this reparative process, so that BMDPC may become an innovative therapeutic strategy option in ARDS and sepsis.

## Methods

### Animal experiments

This study was approved by the Institutional Review Board for the care of animal subjects (University of Heidelberg, Mannheim, Germany & the Regional Council of Karlsruhe, Germany). All animals received humane care in compliance with the “Principles of Laboratory Animal Care” formulated by the National Society for Medical Research and the stipulations of the German Animal Protection Law in its current version.

LPS-induced ARDS was performed as previously described [15]. Briefly, specific pathogen free male Wistar rats housed in standard conditions with food and water ad libitum were anaesthetized by intraperitoneal (IP) injection of ketamine hydrochloride (50 mg/kg) and xylazine (2 mg/kg). Anaesthesia was maintained with intravenous ketamine via an infusion pump (Braun Perfusor Secura ft, B. Braun Melsungen AG, Melsungen, Germany) at an initial rate of 20 mg/kg/h. The level of anaesthesia was assessed by pinching the paw and tail throughout the experiments. The femoral artery was cannulated with a polyethylene catheter (PE-50, neoLab Heidelberg, Germany) for multiple arterial blood collection. Subsequently, a catheter system (AeroProbe, Trudell Medical International, Ontario, Canada) was placed in the trachea for nebulization of lipopolysaccharide (LPS) or saline solution. The operating system (LABneb, Trudell Medical International, Ontario, Canada) exerts a pressure of 60 psi and applies a short puff synchronized with the respiration. The catheter system was removed after application of the respective LPS (serotype: E. Coli O55:B5, Sigma, Deisenhofen, Germany) dose [of 25 μg/kg of body weight].

Following the experimental procedure, the animals were observed with regard to weight changes by the examiner of the group and independently thereof by the animal keepers twelve, twenty-four, forty and seventy-two hours after LPS- or LPS plus EPC-administration. At the final stage after seventy-two hours, the animals were euthanized under deep anaesthetic, and the lungs were exposed for removal. The right-hand middle lobe was weighed directly after the final stage and after twenty-four hours in the cabinet drier to determine wet/dry-ratio (W/D ratio), a correlate for quantification of pulmonary edema. Remaining pulmonary lobes and organs were conserved in paraffin. We will refer to the different animal groups in the rest of our manuscript as follows: control group = animals receiving only sodium chloride; non-treatment group = animals with LPS-induced ARDS, which were not administered BMDPC; treatment group = animals with LPS-induced ARDS, which were administered BMDPC.

### Isolation and administration of bone marrow-derived progenitor cells

In order to isolate bone marrow-derived progenitor cells (BMDPC), the femoral bone marrow of about eight-weeks-old Wistar rats has been used; the rats have first been narcotized with Isoflurane and then euthanized by cervical dislocation. The cell cylinder was filtered through a 40 μm filter, and the mononuclear cells were isolated by density centrifugation. The vitality test of the cell suspension was carried out with trypan blue staining. BMDPC were isolated from the bone marrow cell suspension by positive selection for CD133 by microbeads associated with monoclonal anti-CD133-antibodies (Miltenyi Biotech). For the CD133-microbead column 2 × 10^8^ bone marrow mononuclear cells were used, and the magnetic column was applied according to the manufacturer’s instructions. Directly after LPS injection, the animals were administered under anaesthesia via the tail vein either a 1x10^6^ suspension of CD133^+^-cells dissolved in 1 ml of sodium chloride 0.9% or only 1 ml sodium chloride 0.9% for the control group.

### Histopathologic evaluation

Lung biopsies were performed at the lower lobes (left and right) in all animals, fixed with 4% non-buffered formalin for 24 hours, parafinized and sectioned for subsequent staining with hematoxylin and eosin, CD45 (Santa Cruz, Heidelberg, Germany), ICAM-1 (R&D Systems, Wiesbaden-Nordenstadt, Germany) and VCAM-1/CD106 (BD Biosciences, Heidelberg, Germany) for histological assessment using light microscopy.

### Endothelial Outgrowth Cell (EOC) Assay

BMDPC were isolated from the bone marrow cell suspension by positive selection for CD133 by microbeads associated with monoclonal anti-CD133-antibodies as described above. Cells were suspended in growth medium (Medium 207), placed on fibronectin-coated dishes and incubated for 3 weeks as described earlier [36]. The medium was replenished every 3 days. Staining with CD133-FITC (Abcam, Cambridge, UK) and CD31-FITC (BD Biosciences, Heidelberg, Germany) was performed to assess endothelial character via phase contrast microscopy.

### BMDPC homing assay

BMDPC were isolated from the bone marrow cell suspension by positive selection for CD133 by microbeads associated with monoclonal anti-CD133-antibodies as described above. Cells were stained with DiI (Life Technologies GmbH, Darmstadt, Germany) and administered directly after LPS injection into the animals. After forty-eight hours the animals were sacrificed and their lung tissue analysed.

### Enzyme-linked immune-sorbent assay (ELISA)

The concentrations of vascular endothelial growth factor (VEGF) were assessed in bronchoalveolar lavage (BAL) using enzyme-linked immunosorbent assay kits (R&D Systems, Wiesbaden-Nordenstadt, Germany) in triplicate samples. Enzyme-linked immunosorbent assays were performed according to the manufacturer’s instructions.

### Statistical methods

The statistical comparisons were carried out by means of the student t-test. We considered p < 0.05 to be statistically significant.

## Abbreviations

ALI: Acute lung injury
ARDS: Acute respiratory distress syndrome
BMDPC: Bone marrow-derived progenitor cells
ELISA: Enzyme-linked immunosorbent assay
EPC: Endothelial progenitor cells
ICAM-1: Intercellular adhesion molecule-1
LPS: Lipopolysaccharide
VCAM-1: Vascular cell adhesion molecule-1
VEGF: Vascular endothelial growth factor.
